# Intra-rater reliability of the bath ankylosing spondylitis disease activity index (BASDAI) and the bath ankylosing spondylitis functional index (BASFI) in children with spondyloarthritis

**DOI:** 10.1186/1546-0096-10-S1-A45

**Published:** 2012-07-13

**Authors:** Michelle Batthish, Alisa Rachlis, Bertha Wong, Samantha Stevens, Michelle Anderson, Brian M Feldman, Ronald M Laxer, Joanne Marcuz, Margaret Reaume, Lynn Spiegel, Kristi Whitney-Mahoney, Shirley ML Tse

**Affiliations:** 1 The Hospital for Sick Children, University of Toronto, Toronto, ON, Canada

## Purpose

Juvenile spondyloarthritis (JSpA), referred to as enthesitis-related arthritis (ERA) sub-type under the International League of Associations for Rheumatology (ILAR) classification of juvenile idiopathic arthritis (JIA), is characterized by inflammation in the joints and entheses. Axial involvement is uncommon at presentation but may develop in the second decade of life. Several instruments assessing disease activity in ankylosing spondylitis have been validated including the BASDAI and BASFI. At this time, there are no disease activity scores for JSpA or ERA. The objective of this study is to measure the intra-rater reliability of the BASDAI and the BASFI in JSpA/ERA.

## Methods

Patients diagnosed with ERA (ILAR criteria) and followed at The Hospital for Sick Children Spondyloarthritis Clinic were included in this study. Patients were excluded if they were unable to understand, speak or write English, if they were less than 6 years of age or greater than 18 years of age. Prospective subjects were consecutively enrolled ( June 2009 to June 2010) and, the patient and/or one parent completed the BASDAI and BASFI at baseline and 2 weeks later (a period during which little change is expected). Intra-class correlation coefficient (ICC) was calculated and values greater than 0.6 were considered indicative of good reliability.

## Results

Forty-eight patients (39 males, 81.2%) were enrolled. The average age at diagnosis was 12.5 years (range, 7.6 to 16.7 years). 41.7% were HLA-B27 positive and 18.8% had a positive family history for ankylosing spondylitis. 52% had involvement of the hip and 40% had radiographic evidence of sacroiliitis. Eight patients dropped out or were excluded due to protocol violation. 40 patients completed both sets of questionnaires. All subjects reported their overall health as “the same” when compared to their baseline visit. The mean BASDAI at baseline was 1.97 ± 1.90 and at 2 weeks was 1.69 ± 1.80 – the reliability was substantial; ICC = 0.74, Bland-Altman limits of agreement (LOA) = 2.4 to -2.8. The mean BASFI at baseline was 0.99 ± 1.49 and at 2 weeks was 0.75 ± 1.00 – likewise the reliability was excellent; ICC = 0.87, Bland-Altman LOA = 1.1 to -1.4. When examining individual questions from the BASDAI and BASFI, the following had the highest ICCs, respectively: “How long does your morning stiffness last from the time you wake up?” and “Doing a full day’s activities, whether it be at home or at work”, ICC = 0.88 each. Meanwhile, from the BASDAI, “How would you describe the overall level of AS neck, back or hip pain you have had?” resulted in the lowest reliability; ICC = 0.57.

## Conclusion

At this time, there is no validated disease activity score for JSpA/ERA. Both the BASDAI and BASFI showed excellent intra-rater reliability in a cohort of ERA patients. Next steps will include the measurement of the construct validity and responsiveness of these tools in JSpA/ERA in order to determine if pediatric rheumatologists can use these as validated disease activity/functional impairment measures in the clinic and in clinical research.

## Disclosure

Michelle Batthish: None; Alisa Rachlis: None; Bertha Wong: None; Samantha Stevens: None; Michelle Anderson: None; Brian M. Feldman: None; Ronald M. Laxer: None; Joanne Marcuz: None; Margaret Reaume: None; Lynn Spiegel: None; Kristi Whitney-Mahoney: None; Shirley M.L. Tse: None.

